# Supplementary material for: Prioritisation of Adverse Drug Events Leading to Hospital Admission and Occurring during Hospitalisation: A RAND Survey
Source: J Clin Med. 2022 Jul 22;11(15):4254. doi: 10.3390/jcm11154254 (PMC9332872; doi:10.3390/jcm11154254)
Supplement: Supplementary file 1 [file jcm-11-04254-s001.zip › Supplement S1_Systematic literature search.pdf]

Supplement S1: Systematic literature search

1.1 Search strategy

Adverse drug events (ADEs) during inpatient stay

Setting hospital (Hospital[mesh]) OR (Hospital Medicine[mesh])) OR (medication systems, hospital[mesh])) OR (pharmacy service, hospital[mesh])) OR (medical records department, hospital[mesh])) OR (hospital administration[mesh])) OR (hospital information systems[mesh])) OR (inpatients[mesh])) OR (hospitalization[mesh])) OR ("Hospital" [All Fields])) OR ("Hospitals" [All Fields])) OR ("inpatient" [All Fields])) OR ("inpatients" [All Fields])) OR ("hospitalized" [All Fields])) OR ("ward" [All Fields])) OR ("wards" [All Fields])) OR ("hospitalised" [All Fields])

AND

ADE/ADR (drug-related side effects and adverse reactions[mesh]) OR (medication errors[mesh])) OR ("adverse drug event*" [All Fields])) OR ("adverse drug reaction*" [All Fields])) OR ("medication error*" [All Fields])) OR ("adverse drug effect*" [All Fields])

AND

Germany (Germany [All Fields] OR german [All Fields] OR (germany[mesh])

NOT

emergency service, hospital[mesh] OR outpatient clinics, hospital[mesh] OR emergency service, psychiatric[mesh] OR psychiatric emergency service[mesh] OR outpatient[Title] OR ambulatory[Title] OR emergency[Title] OR systematic review[Title/Abstract] OR meta analysis [Title/Abstract]

Adverse drug events (ADEs) at hospital admission

Setting hospital admission (("emergency service, hospital"[MeSH Terms] OR "hospitalization"[MeSH Terms] OR "emergency medical services"[MeSH Terms] OR "patient admission"[MeSH Terms] OR "hospital admission"[All Fields] OR "emergency department"[All Fields]))

AND

ADE/ADR (drug-related side effects and adverse reactions[mesh]) OR (medication errors[mesh]) OR ("adverse drug event*" [All Fields]) OR ("adverse drug reaction*" [All Fields]) OR ("medication error*" [All Fields]) OR ("adverse drug effect*" [All Fields])

AND

Germany (Germany [All Fields] OR german [All Fields] OR (germany[mesh]))

1.2 Inclusion and exclusion criteria

ADEs during inpatient stay

Table S1. Inclusion and exclusion criteria for the literature search on inpatient ADEs

	Inclusion criteria	Exclusion criteria
**E1:** Study design	Retrospective or prospective intervention or observational study	○ Review<br>○ Meta-analysis<br>○ Simulation study<br>○ Survey<br>○ Clinical trial
**E2:** Study population	Patients ≥ 18 years with a drug prescription and a hospital stay	Focus on patients with a specific disease/drug intake
**E3:** Setting	Hospital in Germany	○ Psychiatry<br>○ Clinics for TCM or similar<br>○ Outpatient clinics
**E4:** Evaluation	**General analysis of ADEs/ADRs**<br>All occurring ADEs/ADRs were considered and at least one of the following quantitative frequencies was described:<br>(I) the frequencies of ADE-associated drug substances<br>(II) the frequencies of the different types of ADEs, e.g. based on organ classes or ICD-10 codes	○ ADEs in the context of hospital admissions or in the emergency department<br>○ ADEs of specific drugs or classes of drugs<br>○ ADEs due to a single DRP, e.g. interactions, overdose

Abbreviations: DRP = drug-related problem; ICD-10 = International Statistical Classification of Diseases and Related Health Problems, 10th revision; TCM = traditional Chinese medicine; ADEs = adverse drug events; ADRs = adverse drug reactions.

ADEs at hospital admission

Table S2. Inclusion and exclusion criteria for the literature search on ADEs at hospital admission

	**Inclusion criteria**	**Exclusion criteria**
**E1:** Study design	Retrospective or prospective intervention or observational study	○ Review<br>○ Meta-analysis<br>○ Simulation study<br>○ Survey<br>○ Clinical trial
**E2:** Study population	Patients ≥ 18 years admitted to hospital or emergency department	Focus on inpatients or patients with specific disease/drug intake
**E3:** Setting	Hospital admission or emergency department visit in German hospitals	○ Admission to specific ward (e.g. ICU)<br>○ Inpatient setting only
**E4:** Evaluation	**General analysis of ADEs/ADRs**<br>All occurring ADEs/ADRs were considered and at least one of the following quantitative frequencies was described:<br>(I) the frequencies of ADE-associated drug substances,<br>(II) the frequencies of the different types of ADEs, e.g. based on organ classes or ICD-10 codes	○ ADEs during hospital stay<br>○ ADEs of specific drugs or drug classes.<br>○ ADEs due to a single DRP, e.g. interactions, overdose

Abbreviations: DRP = drug-related problem; ICD-10 = International Statistical Classification of Diseases and Related Health Problems, 10th revision; TCM = traditional Chinese medicine; ADEs = adverse drug events; ADRs = adverse drug reactions.

1.3 Results

ADEs during inpatient stay

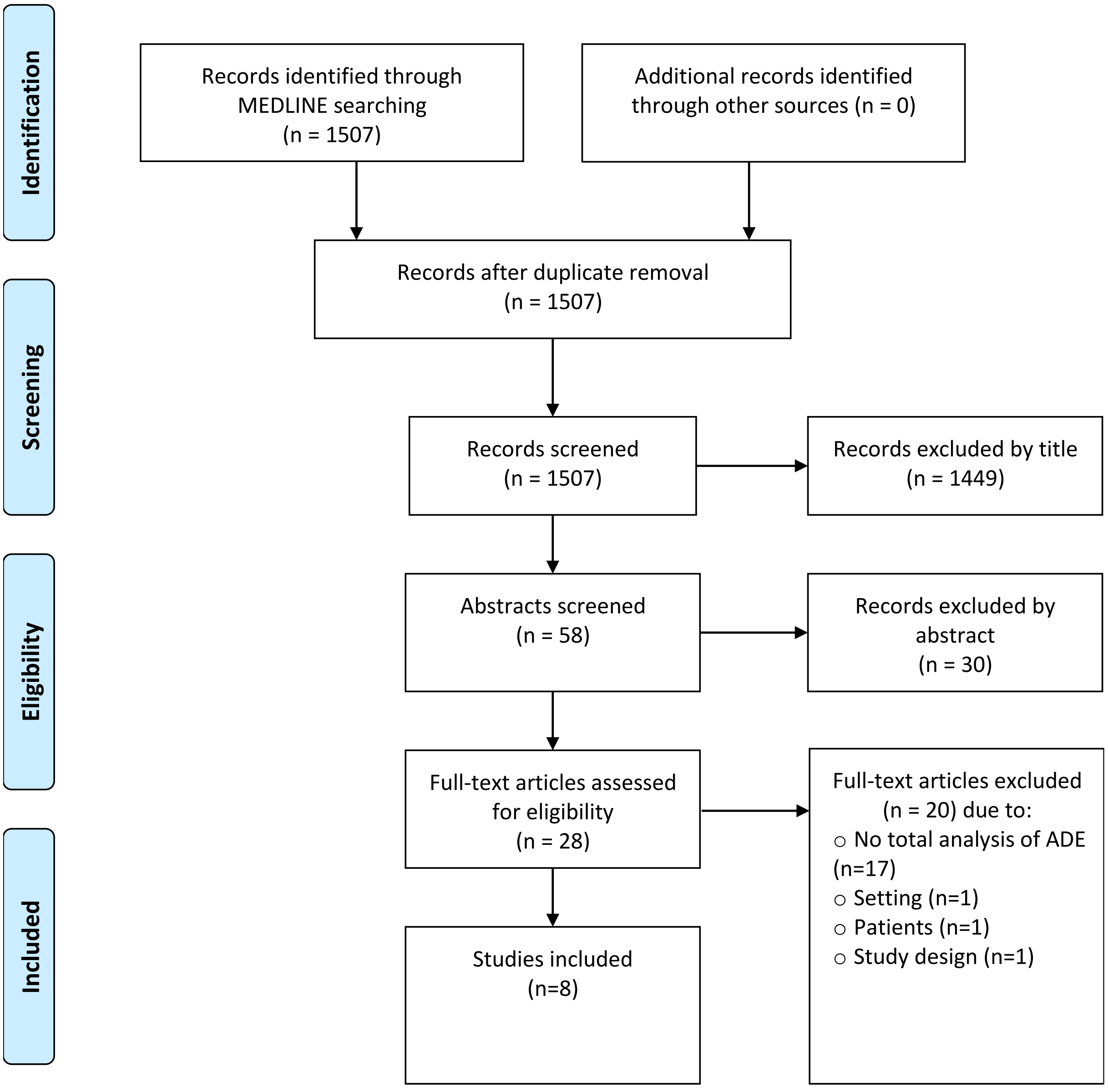

Figure S1. Results of the systematic literature search on in-hospital adverse drug events (ADEs) [1]

ADEs at hospital admission

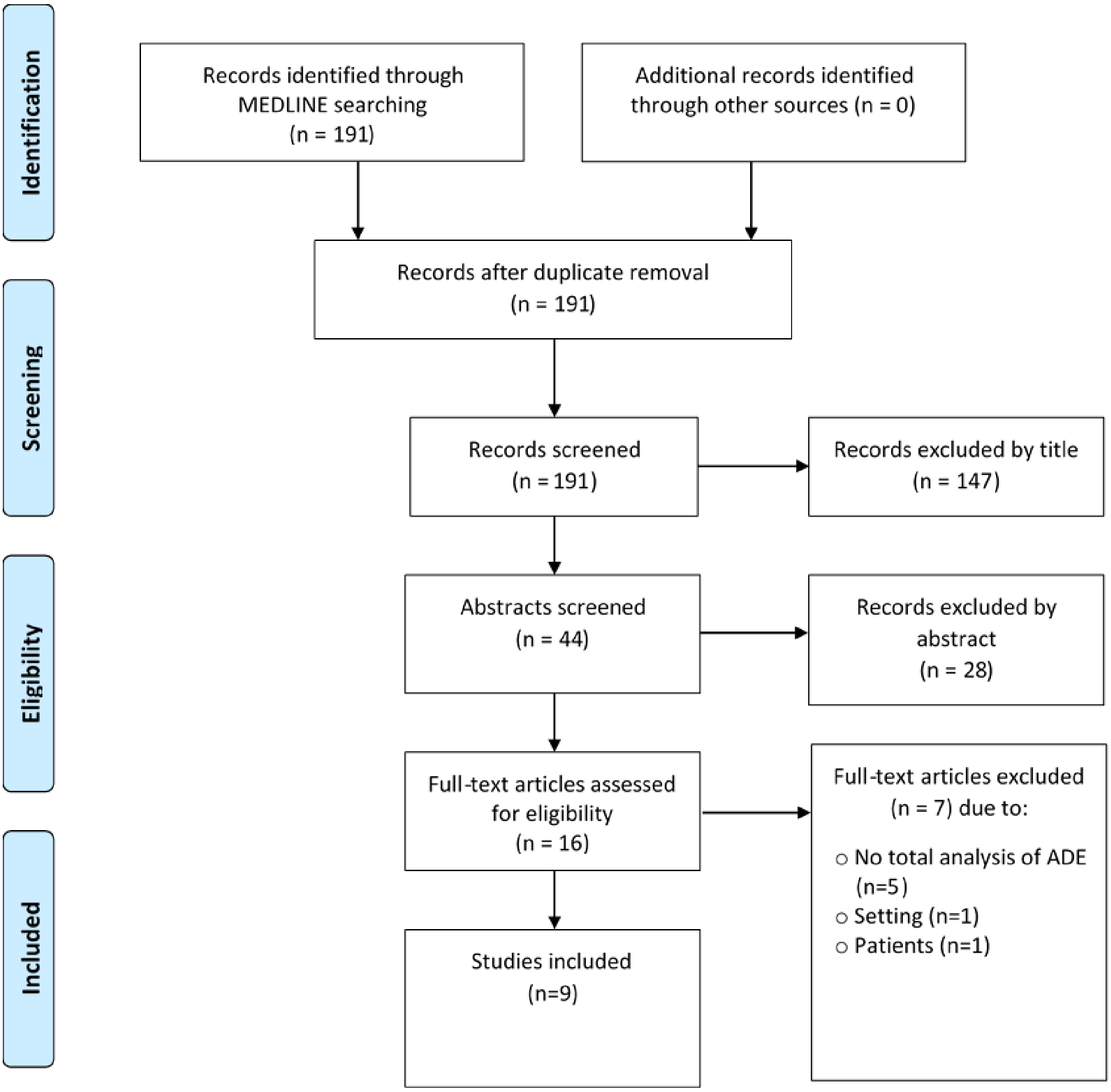

Figure S2. Results of the systematic literature search on adverse drug events (ADEs) at hospital admission [1]

[1] Moher D, Liberati A, Tetzlaff J, Altman DG; PRISMA Group. Preferred reporting items for systematic reviews and meta-analyses: the PRISMA statement. PLoS Med. 2009 Jul 21;6(7): e1000097
