# Supplementary material for: Prioritisation of Adverse Drug Events Leading to Hospital Admission and Occurring during Hospitalisation: A RAND Survey
Source: J Clin Med. 2022 Jul 22;11(15):4254. doi: 10.3390/jcm11154254 (PMC9332872; doi:10.3390/jcm11154254)
Supplement: Supplementary file 1 [file jcm-11-04254-s001.zip › Supplement S2_Interview guide.pdf]

Supplement S2: Interview guide

Translated interview guide

Introduction

Thank you for taking the time to test our RAND procedure for identifying clinically relevant adverse drug events (ADEs) at hospital admission/during hospitalisation*.

Content start

Through the RAND process, we aim to combine the best available scientific evidence with the collective judgement of experts to identify the most important/relevant ADEs during hospital stay or on admission. Based on the results of the first two rounds, risk constellations for the most important ADEs will be assessed in rounds 3 and 4.

Comprehension of the assessment criteria

1. How did you generally deal with the assessment criteria? Did you understand them?
2. Did you have problems with "intermediate" levels 2 and 3? Did you use them?
3. Did the assessment of "seriousness" help you to assess "overall importance"?
4. Did the assessment of "drug-relatedness" help you to assess "overall importance"?

In the following, I would like to discuss each of the assessment criteria with you in detail.

5. What considerations did you make when assessing "overall importance"? What challenges did you face during the assessment?
6. What considerations did you make when assessing "seriousness"? What challenges did you face in the assessment?
7. What considerations did you make when assessing "drug-relatedness"? What challenges did you face in the assessment?

Comprehension of the adverse drug events:

1. Which events were unclear? Please name the corresponding events and the respective ambiguities.
   Could a fine-grained division of the event contribute to more clarity?
2. Did you base your evaluation on an exemplary event from the event category or did you use all events of a category for your evaluation?

Assessment of the evidence report

1. How helpful was the attached evidence report?
2. What further information would you like to see?

Final question

Do you have any further suggestions regarding our RAND process?

*dependent on panel
