# Supplementary material for: Prioritisation of Adverse Drug Events Leading to Hospital Admission and Occurring during Hospitalisation: A RAND Survey
Source: J Clin Med. 2022 Jul 22;11(15):4254. doi: 10.3390/jcm11154254 (PMC9332872; doi:10.3390/jcm11154254)
Supplement: Supplementary file 1 [file jcm-11-04254-s001.zip › Supplement S3_Excluded adverse drug events (ADEs).pdf]

Supplement S3: Excluded adverse drug events (ADEs)

The following ADEs were excluded due to

(1) Complications from surgical or medical procedures, poisoning, ADEs involving only children or pregnant women, and ADEs resulting from underuse:

- Laryngospasm
- Damage to the embryo/fetus/newborn by medicines, drugs or toxins
- Other and unspecified disorders/diseases due to medicinal products
- Poisoning due to medicines
- Poisoning or harmful use of medicines or other substances
- Uncontrolled pain
- Complications due to anesthesia
- Complications of surgical procedures and medical treatment

(2) Unlikeliness to lead to hospital admission or worsen acutely or to occur during a hospital stay of average duration (7 days)

- Obstipation
- Polyneuropathy*
- Osteoporosis and other osteopathies
- Diseases of the connective tissue
- Obesity
- Hypofunction and other disorders of the pituitary gland
- Hypertrophy of the mammary gland
- Sexual dysfunction not caused by an organic disorder or disease
- Pruritus

* This event was only excluded in panel 2 (hospital stay)

Note: The ADEs ‘falls’ and ‘fractures’ were assessed separately in panel 2, whereas ‘fall (injuries)’ was rated as one ADE in panel 1.
