# Supplementary material for: Prioritisation of Adverse Drug Events Leading to Hospital Admission and Occurring during Hospitalisation: A RAND Survey
Source: J Clin Med. 2022 Jul 22;11(15):4254. doi: 10.3390/jcm11154254 (PMC9332872; doi:10.3390/jcm11154254)
Supplement: Supplementary file 1 [file jcm-11-04254-s001.zip › Supplement S4 Rating rounds/Supplementary Table S1_Rating rounds 1.pdf]

Supplementary Table S1: Expert ratings in the first round of the RAND survey

Table S1 Expert ratings of the first round

	Panel 1: Adverse drug events at hospital admission							Panel 2: Adverse drug events during hospital stay						
	**Overall Importance**					**Seriousness**	**Drug-relatedness**	**Overall Importance**					**Seriousness**	**Drug-relatedness**
	Number of participants with score							Number of participants with score						
	1	2	3	4	Median	Median	Median	1	2	3	4	Median	Median	Median
Decompensated heart failure	2	1	5	5	3	3	2	0	7	3	2	2	3,5	2
Tachycardia - supraventricular	0	5	5	3	3	2	2	0	5	6	1	3	2	2
Tachycardia - ventricular	0	0	4	9	4	4	3	0	1	4	7	4	4	3
Bradycardia (requiring treatment)*	0	1	5	7	4	3	3	0	1	8	3	3	3	3
Hypertensive crisis	0	5	7	1	3	2	2	1	4	4	3	3	3	2
Hypotension (requiring treatment)*	0	2	8	3	3	2	2	1	2	4	5	3	2	3
Syncope and collapse	0	0	7	6	3	2	3	0	4	3	5	3	3	3
Acute coronary syndrome	2	6	4	1	2	4	1	3	5	1	3	2	4	1
Cerebral infarction	2	5	3	3	2	4	1	3	5	2	2	2	4	1
Thrombosis	0	5	7	1	3	3	2	1	6	1	4	2	3	2
Bleeding outside the gastrointestinal tract	0	0	6	7	4	4	3	0	1	4	7	4	4	3
Anaemia	1	2	6	4	3	2	2	0	7	3	2	2	2	2
Thrombocytopenia	0	3	8	2	3	3	2	0	4	5	3	3	2	2

Agranulocytosis and neutropenia	0	2	5	6	3	3	3	0	1	4	7	4	3,5	3,5
Bleeding/perforations of the upper gastrointestinal tract	0	0	4	9	4	3	3	0	2	3	7	4	3	3
Gastrointestinal ulcers without bleeding/perforations	0	1	5	7	4	2	3	0	5	3	4	3	2	3
Gastroenteritis, colitis	0	7	3	3	2	2	2	1	5	2	4	2,5	3	2
Acute pancreatitis	3	8	2	0	2	3	2	2	2	7	1	3	3,5	2
Nausea and vomiting (requiring treatment)*	0	7	5	1	2	1	2	1	5	2	4	2,5	2	3
Diarrhoea (requiring treatment)* (excl. gastroenteritis, colitis)	0	7	4	2	2	2	2	0	7	4	1	2	2	2,5
Intestinal obstruction (ileus)	0	4	7	2	3	3	2	0	3	4	5	3	3	2,5
Seizure	0	7	2	4	2	3	2	0	4	3	5	3	3,5	2
Extrapyramidal disorders	0	2	5	6	3	2	3	0	1	6	5	3	3	3,5
Polyneuropathy	2	8	2	1	2	2	2	-	-	-	-	-	-	-
Headache (requiring treatment)* (excl. migraine)	1	9	2	1	2	1	2	3	6	2	1	2	1,5	2
Delirium	0	0	4	9	4	3	3	0	0	4	8	4	3,5	3
Dizziness (requiring treatment)*	0	2	8	3	3	2	3	0	4	5	3	3	2	3
Hallucinations	0	4	4	5	3	2	2	2	2	5	3	3	3	2
Serotonin syndrome	0	0	4	9	4	3	4	1	2	1	8	4	3	4
Somnolence	0	2	5	6	3	2	3	-	-	-	-	-	-	-
Visual disorders (requiring treatment)* (excl. glaucoma)	3	7	3	0	2	2	2	3	6	2	1	2	2	2
Glaucoma attack	3	5	2	3	2	3	2	0	5	6	1	3	2,5	2,5

Toxic damage to the inner ear (excl. vertigo)	0	6	4	3	3	3	2	0	2	6	4	3	3	3
Exacerbation of asthma (excl. pneumonia)**	1	6	4	2	2	2	2	0	5	4	2	3	3	2
Exacerbation of chronic obstructive pulmonary disease (COPD) (excl. pneumonia)	4	3	4	2	2	2	2	2	5	4	1	2	3	2
Pulmonary oedema	1	8	3	1	2	3	2	1	6	2	3	2	3	2
Pneumonia (excl. exacerbation of asthma/COPD)**	3	7	3	0	2	3	1	3	4	3	1	2	3	2
Interstitial lung diseases	4	3	3	3	2	3	2	1	7	2	2	2	3	2
Respiratory depression	0	1	6	6	3	4	3	1	0	6	5	3	3,5	3,5
Fall (injuries) *(Panel 1)/*<br>Falls (requiring treatment)* (excl. fractures) *(Panel 2)*	0	0	5	8	4	3	3	0	2	4	6	3,5	3	3
Fractures	-	-	-	-	-	-	-	1	2	5	4	3	4	2,5
Acute gout attack	1	5	7	0	3	2	2	0	7	2	3	2	2	2
Rhabdomyolysis	0	2	4	7	4	3	3	0	1	2	9	4	4	3,5
Myopathy (excl. rhabdomyolysis)	0	4	8	1	3	2	2	-	-	-	-	-	-	-
Uncontrolled hyperglycaemia	1	2	7	3	3	2	2	0	4	3	5	3	3	2
Hypoglycaemia (requiring treatment)*	0	0	3	10	4	4	4	0	1	2	9	4	3	4
Thyroid dysfunction (requiring treatment)*	2	6	3	2	2	2	2	2	3	3	4	3	2	3
Disorders of the adrenal cortex	0	3	6	4	3	3	3	0	4	6	2	3	3	3
Exsiccosis/dehydration (requiring treatment)*	1	1	6	5	3	3	3	1	2	4	5	3	3	3
Hypernatraemia (requiring treatment)*	3	5	5	0	2	3	2	2	2	6	2	3	3	2

Hyponatraemia (requiring treatment)*	0	3	7	3	3	3	3	0	3	4	5	3	3	3
Lactate acidosis (requiring treatment)*	0	5	6	2	3	3	2	0	2	6	4	3	4	2
Alkalosis (requiring treatment)*	3	8	2	0	2	2	2	1	6	4	1	2	2	2
Hyperkalaemia (requiring treatment)*	0	0	7	6	3	3	3	0	1	3	8	4	3	3
Hypokalaemia (requiring treatment)*	0	0	8	5	3	3	3	0	2	4	6	3,5	3	3
Hypercalcaemia (requiring treatment)*	2	7	4	0	2	2	2	0	4	6	2	3	3	2
Acute kidney injury	0	0	0	13	4	3	3	0	0	2	10	4	3	3
Urinary retention	1	3	7	2	3	2	2	1	6	3	2	2	2	2
Liver damage	0	0	5	8	4	3	2	0	0	3	9	4	3	3
Stevens-Johnson syndrome/ toxic epidermolytic necrosis (TEN)	0	1	1	11	4	4	4	2	2	2	6	3,5	4	3
Other allergic skin reactions (requiring treatment)*	0	4	0	9	4	2	2	0	2	6	4	3	2	3
Anaphylactic shock	1	0	2	10	4	4	2	0	2	2	8	4	4	3
Angioedema	0	0	5	8	4	3	3	0	4	4	4	3	4	3
Infections (requiring treatment)* (excl. pseudomembranous colitis)	1	9	2	1	2	3	2	2	3	2	5	3	3,5	2

* Addition 'requiring treatment' only for adverse drug events during hospital stay (panel 2); in panel 1, all ADEs should be considered for assessment as sufficiently severe to warrant hospital admission; ** Missing values in panel 2
