# Supplementary material for: Prioritisation of Adverse Drug Events Leading to Hospital Admission and Occurring during Hospitalisation: A RAND Survey
Source: J Clin Med. 2022 Jul 22;11(15):4254. doi: 10.3390/jcm11154254 (PMC9332872; doi:10.3390/jcm11154254)
Supplement: Supplementary file 1 [file jcm-11-04254-s001.zip › Supplement S4 Rating rounds/Supplementary Table S2_Ratings Round 2_Panel 1.pdf]

Supplementary Table S2: Expert ratings in the second round of the RAND survey panel 1 (hospital admission)

Table S2 Expert ratings in the second round (panel 1)

	Panel 1: Adverse drug events at hospital admission		
	**Overall Importance** Median	**Seriousness** Median	**Drug-relatedness** Median
Prioritised adverse drug events without disagreement			
Acute kidney injury	4	3	3
Hypoglycaemia	4	4	4
Stevens-Johnson syndrome / toxic epidermolytic necrosis (TEN)	4	4	4
Delirium	4	3	3
Serotonin syndrome	4	3	4
Anaphylactic shock	4	4	2
Tachycardia - ventricular	4	4	3
Bleeding outside the gastrointestinal tract	4	4	3
Bleeding/perforations of the upper gastrointestinal tract	4	3	3
Fall (injuries)	4	3	3
Other allergic skin reactions	4	2	2
Rhabdomyolysis	4	3	3
Liver damage	4	3	2
Angioedema	4	3	3
Gastrointestinal ulcers without bleeding/perforation	4	2	3
Bradycardia	4	3	3
Pseudomembranous colitis	4	3	4
Hyperkalaemia	3	3	3
Syncope and collapse	3	2	3
Agranulocytosis and neutropenia	3	3	3
Somnolence	3	2	3
Respiratory depression	3	4	3
Hypokalaemia	3	3	3
Extrapyramidal disorders	3	2	3
Exsiccosis/dehydration	3	3	3
Anaemia	3	3	3

Intestinal obstruction (ileus)	3	3	2
Dizziness	3	2	3
Hallucinations	3	2	2
Hyponatraemia	3	3	3
Disorders of the adrenal cortex	3	3	3
Decompensated heart failure	3	3	2
Hypotension	3	2	2
Thrombocytopenia	3	3	3
Interstitial lung diseases	3	3	2
Uncontrolled hyperglycaemia	3	2	2
Urinary retention (≥ 65 years)	3	2	2
Myopathy (without rhabdomyolysis)	3	2	2
Adverse drug events with disagreement on prioritisation			
Thyroid dysfunction	3	2	2
Toxic damage to the inner ear (excl. vertigo)	3	3	2
Acute gout attack	3	2	2
Thrombosis	3	3	2
Adverse drug events with disagreement on non-prioritisation			
Seizure	2	3	2
Tachycardia - supraventricular	2	2	2
Urinary retention (< 65 years)	2	2	2
Nausea and vomiting	2	1	2
Exacerbation of chronic obstructive pulmonary disease (COPD) (excl. pneumonia)	2	2	2
Non-prioritised adverse drug events without disagreement			
Diarrhoea (excl. gastroenteritis, colitis)	2	2	2
Exacerbation of asthma (excl. pneumonia)	2	2	2
Lactate acidosis	2	3	2
Hypertensive crisis	2	2	2
Glaucoma attack	2	3	2
Cerebral infarction	2	4	1
Polyneuropathy	2	2	2
Headache (excl. migraine)	2	2	2
Visual disorders (excl. glaucoma)	2	2	2
Gastroenteritis, colitis	2	2	2
Acute pancreatitis	2	3	2

Hypercalcaemia	2	2	2
Infections (excl. pseudomembranous colitis)	2	2	2
Pulmonary oedema	2	3	2
Pneumonia (excl. exacerbation of asthma/COPD)	2	3	1
Hypernatraemia	2	3	2
Alkalosis	2	2	2
Acute coronary syndrome	2	4	1

Median = 4 Median = 3 Median = 2 Median = 1

Frequency of assessment patterns:

Overall Importance	Seriousness	Drug-relatedness	Frequency
Prioritised	Prioritised	Prioritised	22
Prioritised	Non-prioritised	Prioritised	5
Prioritised	Prioritised	Non-prioritised	7
Prioritised	Non-prioritised	Non-prioritised	8
Non-prioritised	Prioritised	Prioritised	0
Non-prioritised	Non-prioritised	Prioritised	0
Non-prioritised	Prioritised	Non-prioritised	9
Non-prioritised	Non-prioritised	Non-prioritised	14
