# Supplementary material for: Prioritisation of Adverse Drug Events Leading to Hospital Admission and Occurring during Hospitalisation: A RAND Survey
Source: J Clin Med. 2022 Jul 22;11(15):4254. doi: 10.3390/jcm11154254 (PMC9332872; doi:10.3390/jcm11154254)
Supplement: Supplementary file 1 [file jcm-11-04254-s001.zip › Supplement S4 Rating rounds/Supplementary Table S3_Ratings Round 2_Panel 2.pdf]

Supplementary Table S3: Expert ratings in the second round of the RAND survey panel 2 (hospital stay)

Table S3 Expert ratings in the second round (panel 2)

	Panel 2: Adverse drug events during hospital stay		
	**Overall Importance** Median	**Seriousness** Median	**Drug-relatedness** Median
Prioritised adverse drug events without disagreement			
Rhabdomyolysis	4	4	4
Acute kidney injury	4	3	3
Hypoglycaemia requiring treatment	4	3	4
Acute liver damage	4	3	3
Anaphylactic shock	4	4	3
Delirium	4	3	3
Bleeding outside the gastrointestinal tract	4	4	3
Hyperkalaemia requiring treatment	4	3	3
Agranulocytosis and neutropenia	4	4	4
Tachycardia - ventricular	4	4	3
Bleeding/perforations of the upper gastrointestinal tract	4	3	3
Pseudomembranous colitis	3	3	3
Extrapyramidal disorders	3	3	3
Serotonin syndrome	4	3	4
Stevens-Johnson-syndrome / toxic epidermolytic necrosis (TEN)	4	4	3
Toxic damage to the inner ear (excl. vertigo)	3	3	3
Falls requiring treatment (excl. fractures)	3	3	3
Hyponatraemia requiring treatment	4	3	3
Hypokalaemia requiring treatment	3	3	3
Angioedema	3	4	3
Bradycardia requiring treatment	3	2	3
Respiratory depression	3	4	3
Fractures	3	4	3
Other allergic skin reactions requiring treatment	3	2	3
Syncope and collapse	3	3	3

Thrombocytopenia	3	3	2
Intestinal obstruction (ileus)	3	3	3
Seizure	3	3	2
Lactate acidosis requiring treatment	3	4	2
Hypotension requiring treatment	3	2	3
Uncontrolled hyperglycaemia	3	3	2
Disorders of the adrenal cortex	3	3	3
Exsiccosis/dehydration requiring treatment	3	3	3
Diarrhoea requiring treatment (excl. gastroenteritis, colitis)	3	2	3
Adverse drug events with disagreement on prioritisation			
Gastrointestinal ulcers without bleeding/perforations	3	2	3
Dizziness requiring treatment	3	2	3
Hallucinations	3	3	2
Hyperthyroidism	3	2	2
Tachycardia - supraventricular	3	3	2
Exacerbation of asthma (excl. pneumonia)	3	3	2
Hypernatraemia requiring treatment	3	3	2
Nausea and vomiting requiring treatment	3	2	3
Adverse drug events with disagreement on non-prioritisation			
Urinary retention	2	2	2
Decompensated heart failure	2	4	2
Infections requiring treatment (excl. pseudomembranous colitis)	2	3	2
Acute coronary syndrome	2	4	1
Cerebral infarction	2	4	1
Glaucoma attack	2	3	2
Hypothyroidism	2	2	2
Alkalosis requiring treatment	2	2	2
Non-prioritised adverse drug events without disagreement			
Thrombosis	2	3	2
Interstitial lung diseases	2	3	2
Hypercalcaemia requiring treatment	2	3	2
Hypertensive crisis	2	3	2
Anaemia	2	2	2
Acute pancreatitis	2	3	1

Pulmonary oedema	2	3	2
Gastroenteritis, colitis	2	2	2
Exacerbation of chronic obstructive pulmonary disease (COPD) (excl. pneumonia)	2	3	2
Acute gout attack	2	2	2
Visual disorders requiring treatment (excl. glaucoma)	2	2	2
Pneumonia (excl. exacerbation of asthma/COPD)	2	3	1
Headache requiring treatment (excl. migraine)	2	2	2

Median = 4 Median = 3 Median = 2 Median = 1

Frequency of assessment patterns:

Overall Importance	Seriousness	Drug-relatedness	Frequency
Prioritised	Prioritised	Prioritised	26
Prioritised	Non-prioritised	Prioritised	7
Prioritised	Prioritised	Non-prioritised	8
Prioritised	Non-prioritised	Non-prioritised	1
Non-prioritised	Prioritised	Prioritised	0
Non-prioritised	Non-prioritised	Prioritised	0
Non-prioritised	Prioritised	Non-prioritised	13
Non-prioritised	Non-prioritised	Non-prioritised	8
